# Systematic Review of Naturally Derived Substances That Act as Inhibitors of the Nicotine Metabolizing Enzyme Cytochrome P450 2A6

**DOI:** 10.3390/ijms25158031

**Published:** 2024-07-23

**Authors:** Haralampos Tzoupis, Konstantinos D. Papavasileiou, Stavros Papatzelos, Angelos Mavrogiorgis, Lefteris C. Zacharia, Georgia Melagraki, Antreas Afantitis

**Affiliations:** 1 Department of ChemInformatics, NovaMechanics Ltd., Nicosia 1070, Cyprus; tzoupis@novamechanics.com (H.T.); papavasileiou@novamechanics.com (K.D.P.); papatzelos@novamechanics.com (S.P.); mavrogiorgis@novamechanics.com (A.M.); 2 Department of ChemInformatics, NovaMechanics MIKE, 18545 Piraeus, Greece; 3 School of Life and Health Sciences, University of Nicosia, Nicosia 1700, Cyprus; zacharia.l@unic.ac.cy; 4 Division of Physical Sciences and Applications, Hellenic Military Academy, 16672 Vari, Greece; georgiamelagraki@gmail.com; 5 Division of Data Driven Innovation, Entelos Institute, Larnaca 6059, Cyprus

**Keywords:** cytochrome P450, CYP450 inhibition, natural products, nicotine, replacement therapy, smoking cessation

## Abstract

Tobacco smoking has been highlighted as a major health challenge in modern societies. Despite not causing death directly, smoking has been associated with several health issues, such as cardiovascular diseases, respiratory disorders, and several cancer types. Moreover, exposure to nicotine during pregnancy has been associated with adverse neurological disorders in babies. Nicotine Replacement Therapy (NRT) is the most common strategy employed for smoking cessation, but despite its widespread use, NRT presents with low success and adherence rates. This is attributed partially to the rate of nicotine metabolism by cytochrome P450 2A6 (CYP2A6) in each individual. Nicotine addiction is correlated with the high rate of its metabolism, and thus, novel strategies need to be implemented in NRT protocols. Naturally derived products are a cost-efficient and rich source for potential inhibitors, with the main advantages being their abundance and ease of isolation. This systematic review aims to summarize the natural products that have been identified as CYP2A6 inhibitors, validated through in vitro and/or in vivo assays, and could be implemented as nicotine metabolism inhibitors. The scope is to present the different compounds and highlight their possible implementation in NRT strategies. Additionally, this information would provide valuable insight regarding CYP2A6 inhibitors, that can be utilized in drug development via the use of in silico methodologies and machine-learning models to identify new potential lead compounds for optimization and implementation in NRT regimes.

## 1. Introduction

Tobacco smoking constitutes a significant public health risk worldwide and it is currently estimated that 1.3 billion people worldwide are using tobacco and tobacco products [1]. In Europe alone, based on the data reported by Eurostat, 19.7% of the population is considered a daily smoker [2]. Smoking has been implicated in cardiovascular diseases, respiratory disorders, and various types of cancer [3], as well as other debilitating conditions [4], while extended nicotine exposure in pregnancy has been associated with adverse neurodevelopmental disorders in babies [5,6,7,8]. The clinical data associated with tobacco smoking have led researchers to study the biochemistry of nicotine addiction and develop strategies to mitigate the problem and help people to quit smoking. Many different strategies have been developed regarding smoking cessation, including drug administration to prolong dopamine levels, psychological intervention [9,10,11], Nicotine Replacement Therapy (NRT) [12,13,14,15], or a combination. All these strategies aim at the gradual reduction in nicotine uptake through smoking and, consequently, to complete smoking cessation. NRT regimes appear to be the most effective of all these strategies, mainly due to their accessibility over-the-counter (OTC). One of the drawbacks regarding NRT success rates is poor adherence by smokers [16,17], while in other cases it can be attributed to the different rates of nicotine metabolism in each person by cytochrome P450 (CYP) [18,19]. Since nicotine is the substance that drives addiction to tobacco smoking [7,20,21], its pharmacokinetic and pharmacodynamic behavior has been the major focus for the development of drugs that can mitigate its action.

Nicotine metabolism has been extensively studied over the years, along with the role of cytochrome P450. The results have shown that the high rate of nicotine metabolism in smokers is correlated with increased nicotine uptake [20]. Smokers metabolize the majority of nicotine to cotinine, via the action of the CYP2A6 variant [22,23,24]. The correlation between CYP2A6 activity, nicotine metabolism, and smoking led researchers to investigate the role of various genetic CYP2A6 polymorphisms in nicotine addiction. Individuals that carry polymorphisms resulting in reduced CYP2A6 activity have a lower rate of nicotine metabolism compared to the normal (wild type) and are thus less nicotine dependent and smoke less [25,26]. At the same time, polymorphisms [alleles *1X2A, *1X2B (gene duplications), and *1B] that result in increased CYP2A6 activity compared to normal, have faster nicotine metabolism, causing individuals to smoke more, and lead to higher susceptibility to addiction [27,28,29]. 

**Figure 1:**
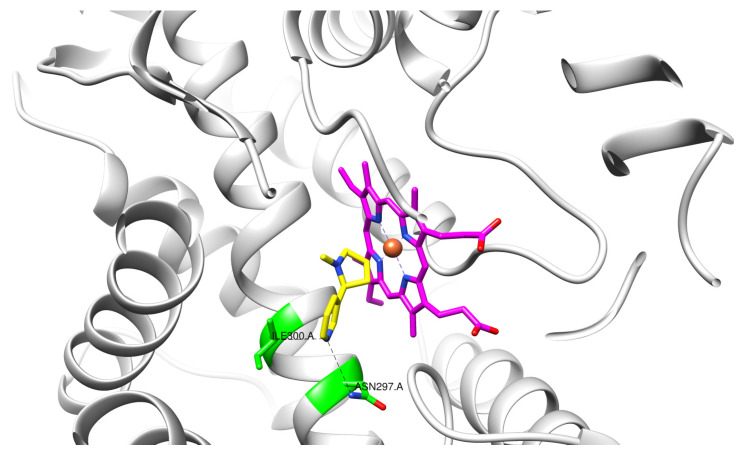
The active site of CYP450 2A6 variant highlighting residues Asn297 and Ile300 that assist in the correct orientation of nicotine (yellow) with the heme moiety (magenta) in the active site. The crystal structure of the CYP450 2A6 with PDB ID 4EJJ [33] was employed.

CYP2A6 has similar structural characteristics to all the enzymes in the cytochrome P450 family. All members of the family contain a single heme moiety at the center of the active site [30,31], despite performing a variety of different functions. For instance, CYP2A6 is involved in the metabolism of foreign compounds that enter the cell, while other members of the family (e.g., CYP5A1) are involved in compound biosynthesis inside the cell [32]. The active site of CYP2A6 also contains residues such as Asn297 and Ile300 (Figure 1) that assist in the orientation of the nicotine alongside the iron atom of the heme [33]. The correct orientation of the ligand inside the active site expedites the catalytic reaction and thus increases the rate of metabolism. The polymorphisms that impact the activity of the enzyme appear to affect the expression of the protein or affect the structural characteristics of the enzyme [20,27,32].

NRT is usually ineffective due to these polymorphisms since NRT is not personalized to account for the different enzyme activities. Thus, inhibitors of the CYP2A6 enzyme have been proposed as a potential strategy for smoking cessation, especially for fast metabolizers, either independently or in conjunction with other NRT strategies [34,35]. A number of studies have tested this type of approach with the use of methoxsalen and coumarin derivatives [36,37,38,39]. The data have shown that methoxsalen has decreased nicotine metabolism in in vivo experiments [37] and in humans, resulting in a 24% decrease in cigarettes smoked compared to the placebo [39]. 

This approach has led researchers to explore all the potential options for developing safe and efficacious CYP2A6 inhibitors. Recent collaborative work has demonstrated that naturally occurring compounds can act as potential CYP2A6 inhibitors [35,40,41,42,43] and consequently, impede nicotine metabolism. The inclusion of dietary products that contain potential CYP2A6 inhibitors could serve as candidates for supplementing NRT regimes by mitigating nicotine uptake. For instance, flavonoids derived from the Gingko Biloba (GB) supplement inhibit the mouse P450 2A5 enzyme, which is the mouse ortholog for human 2A6 [42]. In fact, the naturally derived flavonoids presented similar inhibitory activity to that of cinnamaldehyde, another naturally derived metabolite, that has been shown to inhibit human 2A6 [43]. Natural products include a wide range of compounds, with diverse structures and chemical properties, with the potential to be used as natural P450 2A6 inhibitors. The structure-activity relationship (SAR) of natural compounds as inhibitors of cytochrome P450 2A6 involves understanding how different molecular structures and functional groups impact their inhibitory efficacy. Studies indicate that key interactions include hydrophobic forces, hydrogen bonding, and molecular geometry, which determine binding affinity and selectivity towards CYP2A6 [44,45,46]. Since inhibition of CYP2A6 has been experimentally proven a potential target for smoking reduction or smoking cessation, identifying all the natural compounds with P450 2A6 inhibitory potential is necessary to advance the relevant research. Accordingly, the scope of the systematic review is to identify the extent of current research regarding natural products that can inhibit nicotine metabolism. This review describes the natural products that have been studied as CYP2A6 inhibitors commenting on their action and highlighting their potential as a cost-efficient way to reduce nicotine addiction, either as stand-alone products or in combination with other NRT strategies.

## 2. Materials and Methods

### 2.1. Protocol and Registration

The systematic review was registered on the OSF database [https://osf.io/39e8f/ (accessed on 16 July 2024)] for systematic reviews with the DOI identifier 10.17605/OSF.IO/39E8F. The core of the review was stipulated as follows: “Which natural products have been experimentally investigated as potential CYP2A6 inhibitors?”. The Systematic Reviews and Meta-Analyses (PRISMAs) checklist were applied (Table S1) [47].

### 2.2. Eligibility Criteria

In the current review, we have included research articles and original papers containing chemical compounds that are natural products and have been experimentally tested (in vitro and in vivo) for potential CYP2A6 inhibition. All the publications examined were authored in English and can be readily accessible via public databases. Studies that contain no experimental data on the compounds or present ambiguous data are excluded.

### 2.3. Study Information Sources and Search Terms

All literature searches were performed via PubMed [48] and Scopus [49]. These databases include entries from international peer-reviewed journals and conference papers. The search on the databases was concluded in April 2024. The search terms employed for the search of abstracts, titles, and keywords were “natural products AND CYP2A6” and “natural products AND Cytochrome P450 2A6”.

The above combination of search terms was employed to retrieve all potential hits. By employing a more general search pattern instead of a very specific one (e.g., “natural products AND CYP2A6 AND inhibitor” or “natural products AND CYP2A6 AND inhibitor AND nicotine”), we aimed to incorporate as many publications as possible to avoid missing any relative literature. The broad specificity of the particular approach allows for the inclusion of papers that may contain relative information that otherwise may be discarded. The only drawback of the particular approach is the increased time required for filtering and screening of the results.

### 2.4. Study Selection

**Figure 2:**
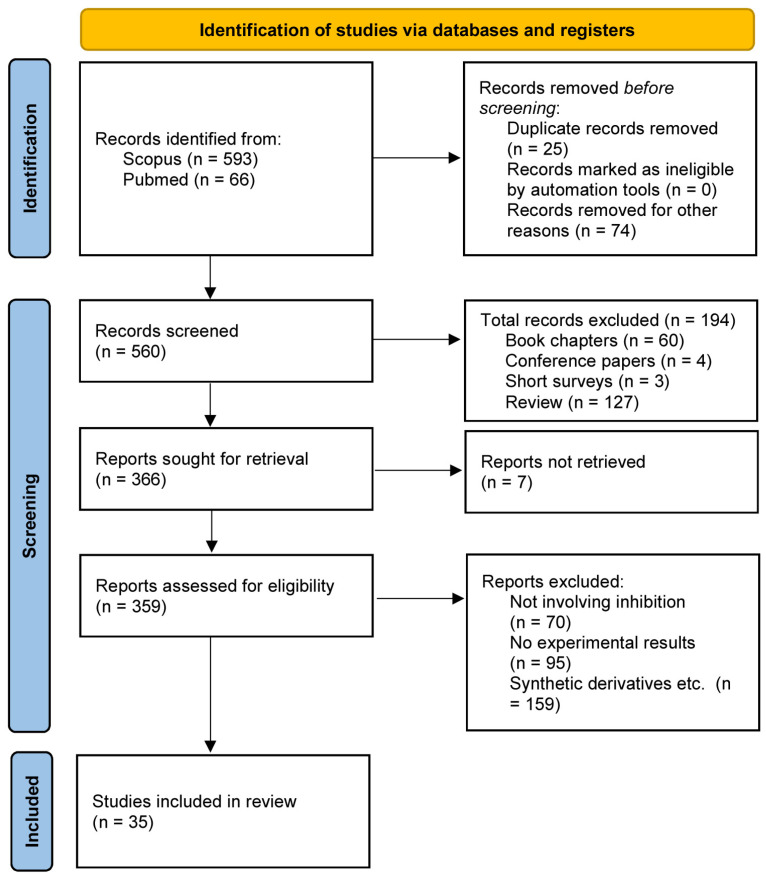
PRISMA 2020 flow diagram of the eligibility assessment process applied in the current systematic review.

The titles and abstracts of papers obtained using the search terms described in Section 2.3 were collected and evaluated. All the publications that did not meet the eligibility criteria were omitted. The remaining papers were examined thoroughly and if they did not provide the necessary information, such as experimental validation, were excluded. The results of the search in both databases yielded a total of 659 literature items. The initial filtering process excluded all the items that did not fall into the category of original articles (e.g., books, reviews, short surveys, editorials, erratum, conference papers, etc.). The initial screening process resulted in 366 papers. The abstracts and titles of these papers were screened to remove any duplicates and papers that did not involve terms such as “inhibition” or did not provide any experimental data. The whole process led to the identification of 35 total papers that were examined in detail. The whole process was carried out by two people working independently to filter the results and is presented in Figure 2.

## 3. Results

Since the identification of CYP2A6 as a potential target for nicotine metabolism inhibition, there has been extensive research in the field. Natural products constitute an extensive library of compounds that cover a great variety of structures and chemical properties. In addition, natural products are a cost-efficient pool of compounds since they can either be easily isolated or they can be incorporated into the diet of a person without having to go under extensive pre-clinical and clinical investigation. The current systematic review has identified and grouped the natural and naturally derived products that have been experimentally tested for inhibition. The search resulted in an initial selection of 659 research articles (593 from Scopus and 66 from PubMed), reporting the effect of natural products on CYP2A6. The PRISMA flowchart (Figure 2) highlights the selection process of the different publications at each stage of the systematic review workflow. Regarding the results from the Scopus database (*n* = 593), the book chapters (*n* = 60), conference papers (*n* = 4), short surveys (*n* = 3), and reviews (*n* = 127) were excluded from the analysis as shown in Figure 2. Table 1 presents the major chemical groups of natural products that have shown CYP2A6 inhibitory activity based on the literature search, as described in Section 2, and Table S2 (Supplementary Information) contains the chemical structures of the different compounds, as well as the reported measured values for different inhibition attributes (IC_50_, K_i_ and % inhibition or remaining activity).

Based on the results of the search, as described above, certain studies were excluded (Table 2). The exclusion criteria for these studies were the absence of information about specific chemical structures that can act as CYP2A6 inhibitors and/or the absence of experimental data for CYP2A6 inhibition. As shown in Table 2, there are studies [61,71,72,73] that have looked at extracts derived from medicinal plants. In these studies, the authors did not consider or report on specific compounds present in the extracts and solely report on the extract’s effects on inhibition. An exception is the study by Rodeiro et al. (2013) [74], which reports the comparison between the plant extract from *Mangifera indica* (mango tree) and pure mangiferin (an ingredient derived from the plant). The rationale for excluding this study was that, even though pure mangiferin presented higher inhibitory activity than the extract, the overall inhibitory activity against CYP2A6 was weak, especially at lower concentrations (<250 μg/mL). Additionally, the study by Budzynska et al. (2016) [61] examined the impact of CYP2A6 inhibitors, like bergapten and 5-methoxypsoralen (5-MOP), on the motor functions of rats but only reports on the level of disruption in motor functions without any experimental information. Finally, Begas et al. (2018) [75] presented the results of a human in vivo study regarding the intake of *Sideritis scardica* decoction and the measured CYP2A6 inhibition.

The results of the literature search have produced a set of 105 compounds (Table 1 and Table S2) that have been experimentally tested as potential CYP2A6 inhibitors. The detailed screening of these papers showed that only 55 of the 105 compounds have demonstrated adequate inhibition of CYP2A6 with IC_50_ < 100 µM and/or Ki < 50 µM (Table S2). Moreover, it is important to note that the researchers in some of these studies have employed natural products as lead compounds in order to test their derivatives as well [65,66]. In the case of Fujita et al. (2001) [66], the authors investigated the activity of 23 organosulfur compounds against CYP2A6. The natural products in the paper comprised 10 compounds and from these, only dipropyl disulfide and diallyl disulfide exhibited potent inhibitory action against CYP2A6 [66]. Similarly, in the study by Qi et al. (2019), from the 17 coumarin derivatives that were investigated, only 7 were natural products [65]. These compounds represent only a tiny fraction of the number of naturally occurring substances. The structural diversity of the examined compounds correlates with the enzymatic function of the cytochrome to metabolize a variety of xenobiotics that enter the cell. In the case of nicotine uptake, the rate of its metabolism is proportional to the addiction caused to the individual. The increased clearance rate of nicotine leads to increased smoking and thus exposes the user to increased health risks.

**Figure 3:**
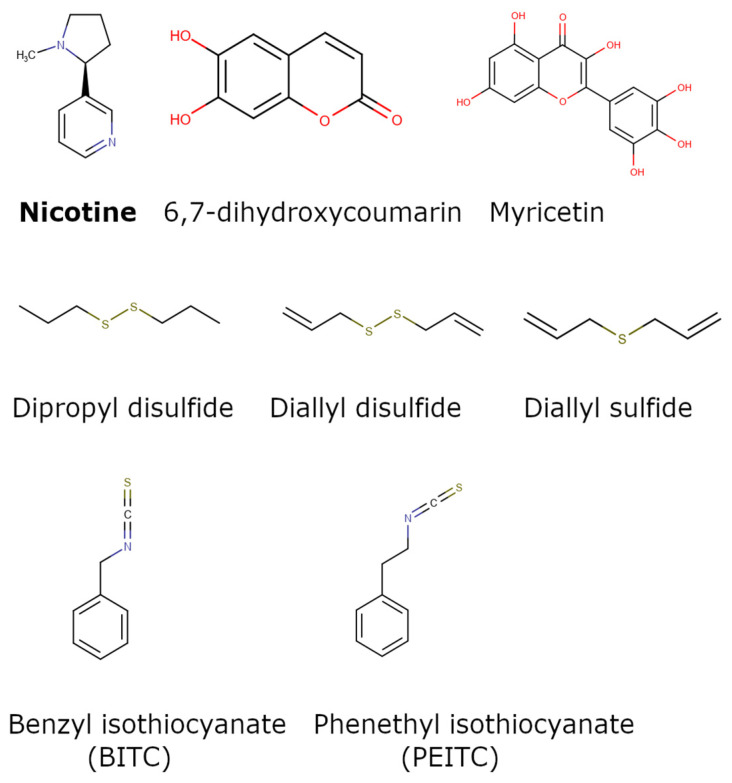
Chemical structures of nicotine and representative CYP2A6 inhibitors: 6,7-dihydroxycoumarin [65], myricetin [58], a flavonoid, dipropyl and diallyl sulfides from the organosulfuric group of compounds [80], and the isothiocyanates benzyl isothiocyanate (BITC) and phenethyl isothiocyanate (PEITC) [50].

Of the natural products examined, flavonoids (*n* = 9), coumarins (*n* = 7), and organosulfur compounds (*n* = 10) comprise the largest number of compounds with inhibitory activity. While organosulfur compounds exhibit characteristically diverse structures [80], flavonoid and coumarin derivatives typically contain at least a single benzopyrone moiety (Figure 3). The presence of the aromatic ring may potentially present similar properties to the ring structure of nicotine and thus could potentially explain the mechanism of action of these analogs. Both myricetin (IC_50_ = 41.4 µM) [58] and 6,7-dihydroxycoumarin (IC_50_ = 0.39 µM, Ki = 0.25 µM) [65] showed competitive binding to CYP2A6, while the coumarin analog presents a very high potency as a CYP2A6 inhibitor. This could be explained by the fact that CYP2A6 is specific for coumarin 7-hydroxylation activity [81]. Furthermore, the analysis carried out through molecular docking by Qi et al. (2019) showed that the binding of 6,7-dihydroxycoumarin closely resembles that of nicotine [65]. Based on their observations, the compound interacts with residues, such as Asn297, which have been highlighted as important to nicotine binding (Figure 1) [33]. These interactions orient the molecules almost perpendicular to the heme moiety of the cytochrome and could compete with nicotine. A similar mode of action may be inferred for myricetin; Tiong et al. (2010) suggested that the number of hydroxyl groups in the flavonoid skeleton increases the binding potential of the compound. In their study, the authors note that myricetin contains six –OH substituents, the highest number from all the flavonoids investigated [58].

Both myricetin and 6,7-dihydroxycoumarin are abundant in natural products. Myricetin, like most flavonoids, is found in various plants, tea, and fruits such as cranberries [82]. On the other hand, 6,7-dihydroxycoumarin is less commonly encountered and is primarily found in medicinal plants [83,84], such as *Phellodendron amurense* var. *wilsonii* [84] and *Fraxinus rhynchophylla* [85]. Similar to flavonoids, organosulfur compounds (Figure 3, middle) can be found in a variety of vegetables and roots (i.e., onion, garlic, broccoli, cabbage, etc.). These compounds are part of the diet of a great portion of the general population and thus can be easily incorporated into NRT strategies. Furthermore, in combination with the isothiocyanate group of compounds (Figure 3, bottom) they can have a potent role in CYP2A6 inhibition. Both isothiocyanate compounds reported present potent binding activity with an inhibitory constant (K_i_) of 4.1 µM for BITC and 0.37 µM for PEITC [50]. The authors of this study state that both compounds inactivate the enzyme and prevent nicotine metabolism up to 80%. The proposed mode of action, as hypothesized in the paper, involves the binding of the compounds or other intermediates to the apoprotein form of CYP2A6 [50].

Many of the compounds reported have a moderate inhibition activity on CYP2A6, while there are compounds that present a weak inhibitory action against the enzyme. These compounds include aromatic aldehydes, such as vanillin and ethyl vanillin [67,86], and the furanocoumarin chalepensin [62], while cinnamaldehyde and benzaldehyde were found to be the most potent inhibitors of microsomal CYP2A6 of the flavoring agents tested in these studies. The two heavier aromatic aldehydes, namely vanillin and ethyl vanillin, exhibited significantly lower potency for microsomal recombinant CYP2A6 inhibition (Table 2 and Table S2). In a similar manner, the 1,4-naphthoquinone derivatives, rhinacanthin H and rhinacanthin I, presented inhibitory activity towards CYP2A6, and the authors have proposed that together with the other rhinacanthin derivatives these compounds may be considered as chemopreventive agents for smokers [63].

Another important consideration regarding natural products in NRT regimes is potential toxicity. Most of the compounds identified in this review do not exhibit high toxicity profiles. In most cases, very high concentrations are required to induce a toxic effect, and therefore, the majority of the compounds presented herein can be considered safe. Out of the 55 compounds identified, tryptamine has been shown to be potentially cytotoxic at concentrations found in many fermented foods (e.g., fish sauce, cheese) [87,88]. Safrole (Table S2) has also been reported to be moderately toxic in in vivo experiments and possibly carcinogenic to humans [89]. Moreover, cannabidiol and its derivatives have demonstrated cytotoxic effects on mouse Leydig cells [90], impact hepatic functions [91], and cause fertility issues [92]. In contrast, the alkaloid humantenine (Table S2), which is extracted from the *Gelsemium* spp. and employed in herbal remedies, exhibits increased toxicity [93]. On the other hand, coumarins, such as 8-methoxypsoralen (xanthotoxin) and bergapten, have shown very low toxicity, while presenting a wide variety of medicinal uses (e.g., anticancer, anti-inflammatory, and antimicrobial activity) [94,95]. Out of the flavonoid compounds, kaempferol (Table 1 and Table S2) has been identified to have potentially minor adverse effects on the liver in high concentrations [96,97]. Vanillin presented a favorable pharmacological profile, similar to 8-methoxypsoralen (xanthotoxin) with low toxicity [96,98], while showing the ability to inhibit or reduce the mutagenesis process initiated by xenobiotics [99].

## 4. Conclusions

Considering the studies mentioned in Section 3 and their findings, it is evident that natural products can offer a rich source of chemically diverse compounds. Based on the various experimental and cheminformatics tools, it is possible to investigate the potential action of natural products for CYP2A6 inhibition. Most approaches for smoking cessation have focused on developing de novo potent inhibitors of CYP2A6 and have utilized only a few naturally derived compounds as scaffolds for developing novel inhibitors. Identifying CYP2A6 inhibitors of natural origin is a cost-efficient way for the development of new products to be incorporated as enhancers of current NRTs, with the aim of increasing success rates. Most of the natural products can be marketed as food supplements and/or as effective medication with increased efficacy. Another benefit of natural products is their potential to serve as medication supplements, as they are associated with fewer side effects compared to current smoking cessation medicines like bupropion.

Moreover, the development of natural product libraries is presenting researchers access to thousands of compounds and scaffolds that can be easily isolated and employed in drug development. In this context, cheminformatic tools can be used to identify novel 2A6 inhibitors and screen the existing natural product databases for potential nicotine metabolism inhibitors. This is deemed necessary given that the current approach of testing natural/plant extracts one by one in the lab is time-consuming and costly. Subsequently, the identified compounds as the most suitable candidates capable of modulating nicotine consumption can be extensively tested in vitro and in vivo. In fact, there have been studies that employed natural compounds as scaffolds to synthesize potential CYP2A6 inhibitors [36,44,65,66,100]. Similarly, the structural basis of the naturally occurring coumarins provided insights into how modifications can improve efficacy in facilitating smoking cessation [36]. In all these cases, the reason behind choosing natural products as scaffolds was the favorable pharmacological profiles of the compounds, such as flavones and coumarins. Additionally, the candidate molecules can be utilized for further preclinical research as NRT enhancers, either in the form of food, dietary supplements, and/or medication, thus increasing NRT adherence and success rates. However, it is important to note that attention must be given to the toxicity profiles of the candidate molecules. As with most chemical substances, natural products can have harmful effects on human cells that are mediated by the intake concentration and the compound’s structure. Several natural products that have been explored for smoking cessation are known to inhibit CYP2A6, such as certain constituents found in grapefruit and other citrus fruits. These compounds can influence the metabolism of nicotine, potentially aiding in smoking cessation by prolonging nicotine’s effects. Nevertheless, there may be potential interactions with other medications, leading to adverse effects due to altered drug metabolism. While natural products in most food sources and traditional herbal remedies are generally considered safe, their potential to cause toxicity at higher doses or through prolonged use cannot be overlooked. This is particularly relevant when these natural products are used concurrently with other medications, as they might lead to unexpected drug interactions and side effects.

## Figures and Tables

**Table 1:** Major chemical groups of natural products with CYP2A6 inhibition.

Group Name	Scaffolds/Representative Structure	No. of Compounds	References
Isothiocyanates	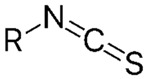	**3**	[50,51]
Terpenes	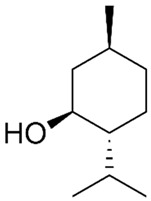	**2**	[52,53]
Alkaloids	Diverse chemical group of organic nitrogen-containing bases	**5**	[52,53,54,55,56]
Flavonoids	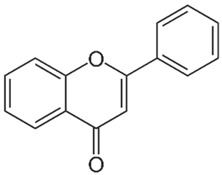	**32**	[57,58,59]
Furanocoumarins/psoralen	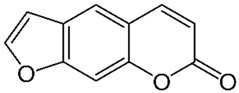	**4**	[60,61,62]
1,4-naphthoquinones	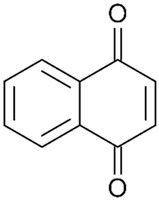	**5**	[63]
Propylamines	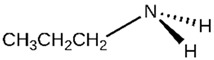	**1**	[60]
Triazoles	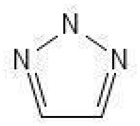	**1**	[64]
Coumarins	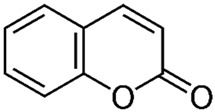	**20**	[61,65]
Thiophenes	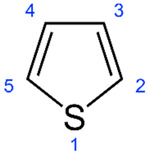	**3**	[59]
Hirsutinolides	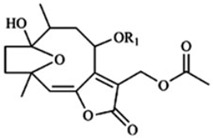	**4**	[59]
Organosulfur compounds	Diverse group that contains structures with at least one –S atom connected to carbon atoms	**23**	[66]
Benzaldehydes	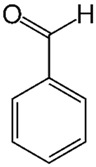	**2**	[53,67]
Cannabidiol derivatives	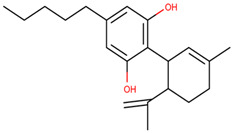	**4**	[68,69]
Benzodioxoles	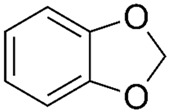	**1**	[70]

**Table 2:** Studies excluded from the analysis.

	Reference	Exclusion Criteria
1	[71]	Study reports only results from Khat ethanol extract.
2	[72]	Report about *Aconiti Lateralis Radix Praeparata* extract (in combination with red ginseng).
3	[73]	Study of CYP2A6 inhibition from the extracts of *Hibiscus sabdariffa*. No report of chemical structures and related data.
4	[61]	The specific study contains data only on in vivo modulation with no inhibitory information on the substances xanthotoxin/bergapten and umbelliferone
5	[74]	Study reporting the comparison between the plant extract from the mango tree and mangiferin (an ingredient derived from the plant) isolated and purified. Weak inhibition results
6	[75]	Study reporting the effect of a decoction from *Sideritis scardica* with no other inhibitory data provided.
7	[76]	Study presents data from mouse experiments but does not report any inhibitory data for CYP2A6 in the form of % inhibition, IC_50_ or K_i_.
8	[77]	Study reports the CYP2A6 inhibition by Danhong injection (DHI) extract with no information on specific compounds that may act as inhibitors.
9	[78]	Study reports the inhibition of CYP2A6 from tropical medicinal herbs with no information on active ingredients.
10	[79]	Study reports the effect of a decoction from *Sutherlandia frutescens* but provides no information on specific compounds with inhibitory activity towards CYP2A6.

## Supplementary Materials

The following supporting information can be downloaded at: https://www.mdpi.com/article/10.3390/ijms25158031/s1. References [43,50,51,52,53,54,55,56,57,58,59,60,62,63,64,65,66,67,68,69,70,101,102,103,104,105,106,107,108,109,110,111,112,113,114] belongs to data in supplementary file.
