# Supplementary material for: Systematic Review of Naturally Derived Substances That Act as Inhibitors of the Nicotine Metabolizing Enzyme Cytochrome P450 2A6
Source: Int J Mol Sci. 2024 Jul 23;25(15):8031. doi: 10.3390/ijms25158031 (PMC11312336; doi:10.3390/ijms25158031)
Supplement: Supplementary file 1 [file ijms-25-08031-s001.zip › ijms-3106935-supplementary.pdf]

Systematic review of naturally derived substances that act as inhibitors of the nicotine metabolizing enzyme cytochrome P450 2A6

Haralampos Tzoupis[1], Konstantinos D. Papavasileiou,[1,2] Stavros Papatzelos,[1] Angelos Mavrogiorgis,[1] Lefteris C. Zacharia,[3] Georgia Melagraki[4] and Antreas Afantitis[1,2,5*]

[1] Department of ChemInformatics, NovaMechanics Ltd., Nicosia CY-1070, Cyprus; tzoupis@novamechanics.com
[2] Department of ChemInformatics, NovaMechanics MIKE., Piraeus GR-185 45, Greece; papavasileiou@novamechanics.com
[3] School of Life and Health Sciences, University of Nicosia, Nicosia 1700, Cyprus; zacharia.l@unic.ac.cy
[4] Division of Physical Sciences and Applications, Hellenic Military Academy, Vari 16672, Greece; georgiamelagraki@gmail.com
[5] Division of Data Driven Innovation, Entelos Institute, Larnaca CY-6059, Cyprus; afantitis@novamechanics.com
* Correspondence: afantitis@novamechanics.com

Table of Contents

1. **Table S1:** PRISMA2020 checklist for systematic reviews

2. **Table S2:** Structures of natural products with CYP2A6 inhibition and the reported values. Compounds with IC_{50} > 100 μM and/or K_i > 50 μM are not presented.

Table S1: PRISMA2020 checklist for systematic reviews

Section and Topic	Item #	Checklist item	Location where item is reported
**TITLE**			
Title	1	Identify the report as a systematic review.	page 1
**ABSTRACT**			
Abstract	2	See the PRISMA 2020 for Abstracts checklist.	page 1
**INTRODUCTION**			
Rationale	3	Describe the rationale for the review in the context of existing knowledge.	Pages 1-3
Objectives	4	Provide an explicit statement of the objective(s) or question(s) the review addresses.	Page 4 line 103
**METHODS**			
Eligibility criteria	5	Specify the inclusion and exclusion criteria for the review and how studies were grouped for the syntheses.	Section 2.2 (page 4)
Information sources	6	Specify all databases, registers, websites, organisations, reference lists and other sources searched or consulted to identify studies. Specify the date when each source was last searched or consulted.	Section 2.3 (page 4)
Search strategy	7	Present the full search strategies for all databases, registers and websites, including any filters and limits used.	Section 2.4 (page 4)
Selection process	8	Specify the methods used to decide whether a study met the inclusion criteria of the review, including how many reviewers screened each record and each report retrieved, whether they worked independently, and if applicable, details of automation tools used in the process.	Section 2.4 (page 4)
Data collection process	9	Specify the methods used to collect data from reports, including how many reviewers collected data from each report, whether they worked independently, any processes for obtaining or confirming data from study investigators, and if applicable, details of automation tools used in the process.	Section 2.4 (page 4)
Data items	10a	List and define all outcomes for which data were sought. Specify whether all results that were compatible with each outcome domain in each study were sought (e.g. for all measures, time points, analyses), and if not, the methods used to decide which results to collect.	N/A
	10b	List and define all other variables for which data were sought (e.g. participant and intervention characteristics, funding sources). Describe any assumptions made about any missing or unclear information.	N/A
Study risk of bias assessment	11	Specify the methods used to assess risk of bias in the included studies, including details of the tool(s) used, how many reviewers assessed each study and whether they worked independently, and if applicable, details of automation tools used in the process.	N/A
Effect measures	12	Specify for each outcome the effect measure(s) (e.g. risk ratio, mean difference) used in the synthesis or presentation of results.	N/A
Synthesis methods	13a	Describe the processes used to decide which studies were eligible for each synthesis (e.g. tabulating the study intervention characteristics and comparing against the planned groups for each synthesis (item #5)).	N/A
	13b	Describe any methods required to prepare the data for presentation or synthesis, such as handling of missing summary statistics, or data conversions.	N/A

Section and Topic	Item #	Checklist item	Location where item is reported
	13c	Describe any methods used to tabulate or visually display results of individual studies and syntheses.	N/A
	13d	Describe any methods used to synthesize results and provide a rationale for the choice(s). If meta-analysis was performed, describe the model(s), method(s) to identify the presence and extent of statistical heterogeneity, and software package(s) used.	N/A
	13e	Describe any methods used to explore possible causes of heterogeneity among study results (e.g. subgroup analysis, meta-regression).	N/A
	13f	Describe any sensitivity analyses conducted to assess robustness of the synthesized results.	N/A
Reporting bias assessment	14	Describe any methods used to assess risk of bias due to missing results in a synthesis (arising from reporting biases).	N/A
Certainty assessment	15	Describe any methods used to assess certainty (or confidence) in the body of evidence for an outcome.	N/A
**RESULTS**			
Study selection	16a	Describe the results of the search and selection process, from the number of records identified in the search to the number of studies included in the review, ideally using a flow diagram.	Section 2.4 (page 4) and Result Section Page 5
	16b	Cite studies that might appear to meet the inclusion criteria, but which were excluded, and explain why they were excluded.	Pages 6-7, and Table 2 in Results section
Study characteristics	17	Cite each included study and present its characteristics.	N/A
Risk of bias in studies	18	Present assessments of risk of bias for each included study.	N/A
Results of individual studies	19	For all outcomes, present, for each study: (a) summary statistics for each group (where appropriate) and (b) an effect estimate and its precision (e.g. confidence/credible interval), ideally using structured tables or plots.	Table S2, Supplementary material
Results of syntheses	20a	For each synthesis, briefly summarise the characteristics and risk of bias among contributing studies.	N/A
	20b	Present results of all statistical syntheses conducted. If meta-analysis was done, present for each the summary estimate and its precision (e.g. confidence/credible interval) and measures of statistical heterogeneity. If comparing groups, describe the direction of the effect.	N/A
	20c	Present results of all investigations of possible causes of heterogeneity among study results.	N/A
	20d	Present results of all sensitivity analyses conducted to assess the robustness of the synthesized results.	N/A
Reporting biases	21	Present assessments of risk of bias due to missing results (arising from reporting biases) for each synthesis assessed.	N/A

Section and Topic	Item #	Checklist item	Location where item is reported
Certainty of evidence	22	Present assessments of certainty (or confidence) in the body of evidence for each outcome assessed.	N/A
**DISCUSSION**			
Discussion	23a	Provide a general interpretation of the results in the context of other evidence.	Pages 4-9., Tables 1 and S2
	23b	Discuss any limitations of the evidence included in the review.	Pages 8-10
	23c	Discuss any limitations of the review processes used.	N/A
	23d	Discuss implications of the results for practice, policy, and future research.	Page 9
**OTHER INFORMATION**			
Registration and protocol	24a	Provide registration information for the review, including register name and registration number, or state that the review was not registered.	www.osf.io
	24b	Indicate where the review protocol can be accessed, or state that a protocol was not prepared.	at the osf.io site
	24c	Describe and explain any amendments to information provided at registration or in the protocol.	N/A
Support	25	Describe sources of financial or non-financial support for the review, and the role of the funders or sponsors in the review.	Page 10
Competing interests	26	Declare any competing interests of review authors.	N/A
Availability of data, code and other materials	27	Report which of the following are publicly available and where they can be found: template data collection forms; data extracted from included studies; data used for all analyses; analytic code; any other materials used in the review.	N/A

From: Page MJ, McKenzie JE, Bossuyt PM, Boutron I, Hoffmann TC, Mulrow CD, et al. The PRISMA 2020 statement: an updated guideline for reporting systematic reviews. BMJ 2021;372:n71. doi: 10.1136/bmj.n71

Table S2: Structures of natural products with CYP2A6 inhibition and the reported values. Compounds with IC_{50} > 100 μM and/or K_i > 50 μM are not presented.

No.	Structure	Name	IC_{50} (μM)	K_i (μM)	% inhibition	% remaining activity	Ref.
1	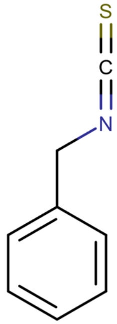	Benzyl isothiocyanate (BITC)	-	4.1±0.8	84		[1]
2	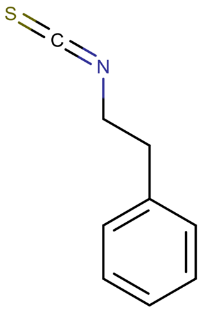	Phenethyl isothiocyanate (PEITC)	-	0.37±0.09<br>1.7±0.28	75<br>-		[1]<br>[2]

3	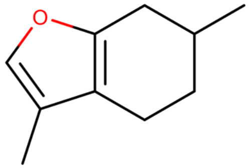	Methofuran	-	0.29±0.05<br>2.0±0.44	95<br>-		[3]<br>[2]
4	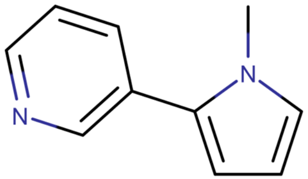	β-nicotyrine	-	1.07±0.02<br>7.5±2.9	51<br>-		[3]<br>[2]
5	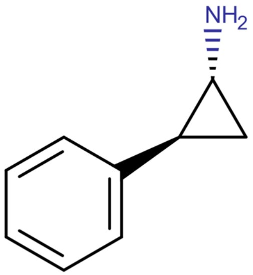	Tranylcypromine	-	0.13±0.02<br>0.08 to 0.2<br>0.05	-<br>-<br>-		[2]<br>[4]<br>[5]

6	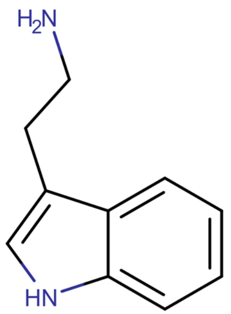	Tryptamine	-	1.7±0.12<br>0.2	-<br>-		[2]<br>[6,7]
7	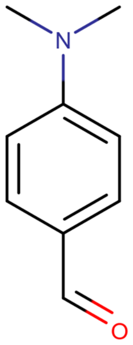	4-dimethylaminobenzaldehyde (DMABA)	-	3.6±0.83	-		[2]
8	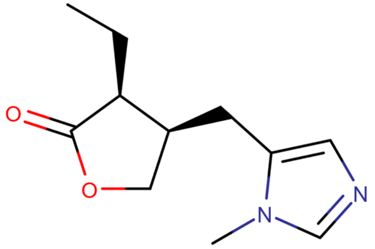	Pilocarpine	-	3.0±0.45<br>1.21±0.51<br>1<br>*1.5*[a]	-<br>-<br>-<br>-		[2]<br>[8]<br>[4,8]<br>[9]

9	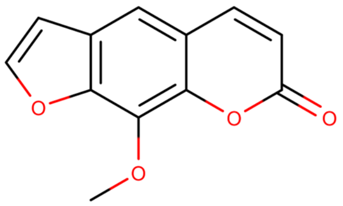	8-Methoxypsoralen, methoxsalen (8-MOP)	-<br>-<br>-<br>0.19±0.05	0.25±0.10<br>0.06<br>1.53±0.01<br>-	-<br>-<br>-<br>-	-<br>-<br>-<br>-	[2]<br>[5]<br>[10]<br>[11]
10	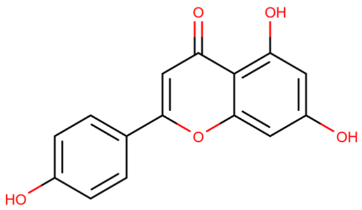	Apigenin	0.90±0.07<br>81.3	0.43±0.17<br>-	53<br>-		[12–14]<br>[15]
11	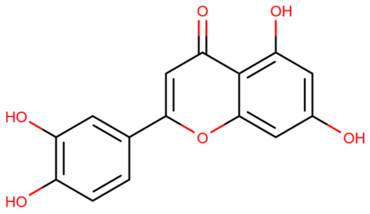	Luteolin	1.38±0.18<br>91.4	0.80±0.06<br>-	-<br>-		[12,13]<br>[15]
12	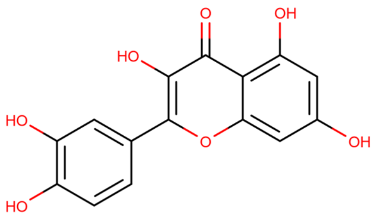	Quercetin	2.66±0.24<br>61.3	1.19±0.27<br>26.70±0.71	-		[13]<br>[15]

13	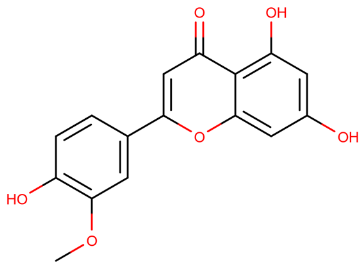	Chrysoeriol	1.14±0.10	0.63±0.12	-		[13]
14	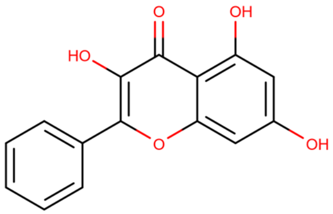	Galagnin	68	37.50±0.24	52		[14,15]
15	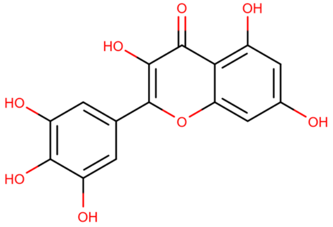	Myricetin	41.4<br>5.26±0.72	33.75±3.89<br>4.06±0.52	-		[15]<br>[16]
16	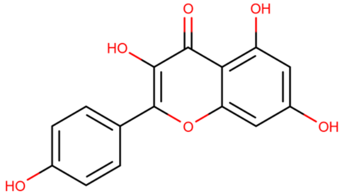	Kaempferol	69.8<br>2.544±0.26	37.59±1.53<br>1.77±0.47	-		[15]<br>[16]

17	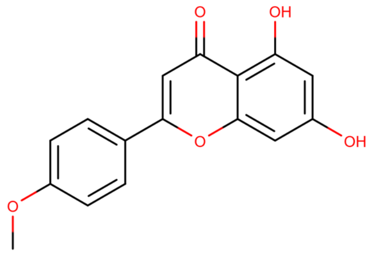	Acacetin	67.6	49.34±6.1<br><br>3	-		[15]
18	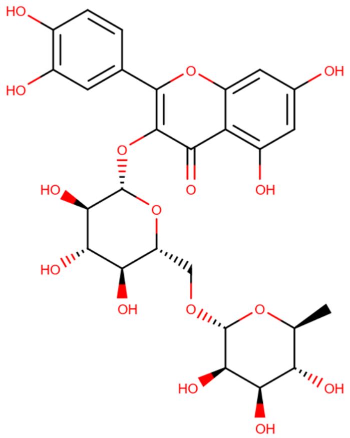	Rutin	78.1	-	-		[15]
19	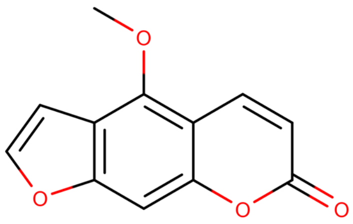	Bergapten/<br>5-methoxypsoralen	-	12.0	-	-	[17]

20	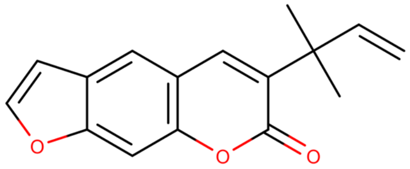	Chalepensin	1.14±0.10	2.6			[18]
21	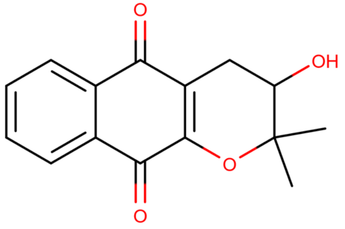	Rhinacanthin-A	1.88±0.13	-	-		[10]
22	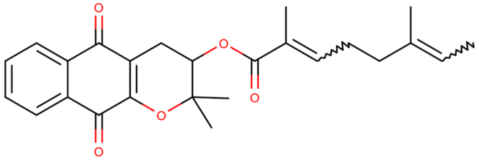	Rhinacanthin-B	2.00±0.03	-	-		[10]
23	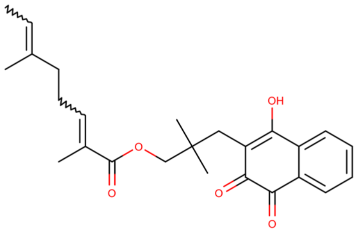	Rhinacanthin-C	5.60±0.94	-	-		[10]

24	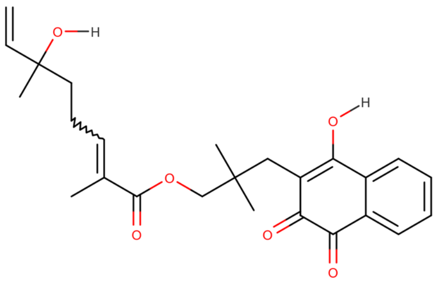	Rhinacanthin-H	5.33±0.76	-	-		[10]
25	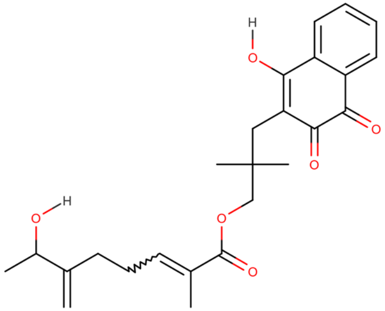	Rhinacanthin-I	5.33±0.76	-	-		[10]
26	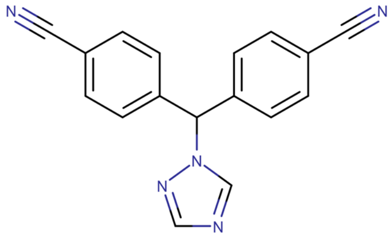	Letrozole	-	4.6±0.1	-		[19]

27	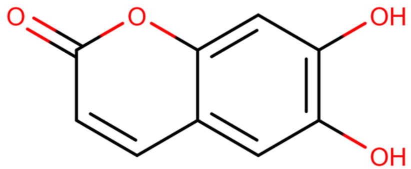	Esculetin	0.39±0.10	0.25±0.13	-	12.3±0.1	[20]
28	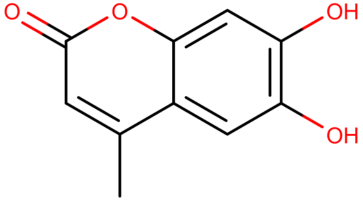	4-methylesculetin	6.70±0.52	3.62±0.77	-	14.5±0.3	[20]
29	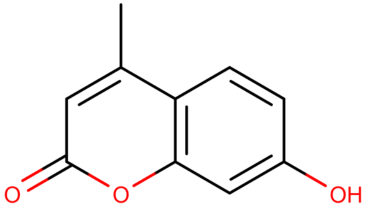	Hymecromone/ 4-methylumbelliferone	64.2±11	36.3±4.6	-	36.3±5.5	[20]
30	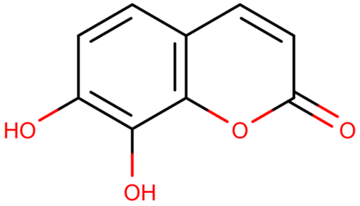	Daphnetin/ 7,8- Dihydroxycoumarin	4.61±1.2	3.02±1.2	-	15.7±0.5	[20]

31	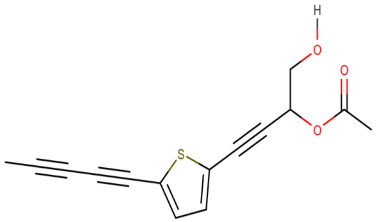	2-(penta-1,3-diyn-1-yl)-5–(4-acetoxy-3-hydroxybuta-1-yn-1-yl) thiophene	6.43±1.29	3.23±0.43	71		[12]
32	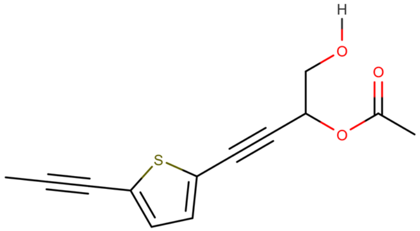	2-(prop-1-inyl)-5–(6-acetoxy-5-hydroxyhexa-1, 3-diinyl) thiophene	4.44±0.14	2.07±0.23	71		[12]
33	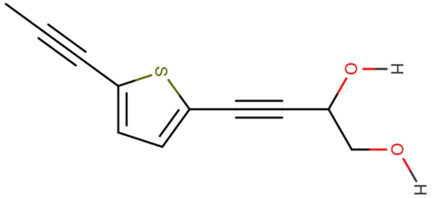	2-(prop-1-inyl)-5–(5, 6-dihydroxyhexa-1,3-diinyl)thiophene	3.90±0.20	1.80±0.18	70		[12]
34	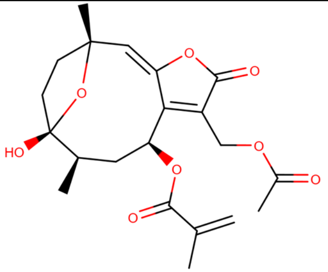	8α-(2-methylacryloyloxy)-hirsutinolide-13-*O*-acetate	22.3±2.5	15.1±2.1	71		[12,13]

35	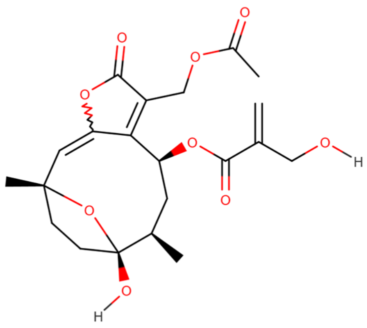	8α-(4-hydroxymethacryloyloxy)-hirsutinolide-13-*O*-acetate	32.7±2.2	15.1±2.1	72		[12,13]
36	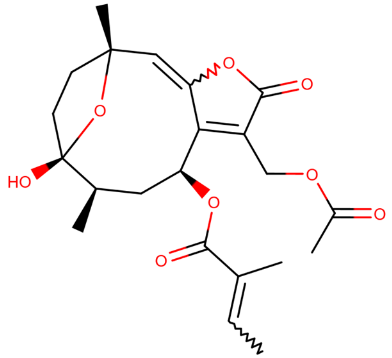	8α-tigloyloxy hirsutinolide-13-*O*-acetate	37.8±3.5	30.6±1.5	78		[12,13]
37	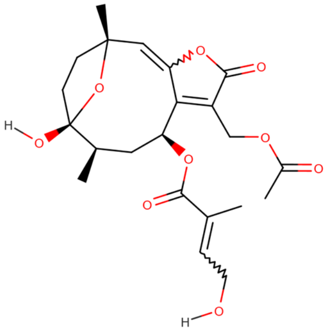	8α-(4-hydroxytigloyloxy)-hirsutinolide-13-*O*-acetate	64.5±5.8	42.3±8.4	77		[12,13]

38	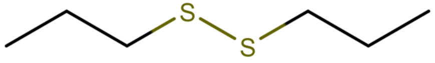	Dipropyl disulfide	-	1.73	-	39.5	[21]
39	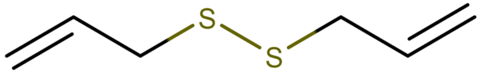	Diallyl disulfide	-	2.13		46.2	[21]
40	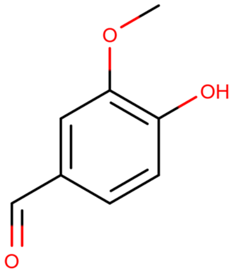	Vanillin	70.0	-	-	-	[22]
41	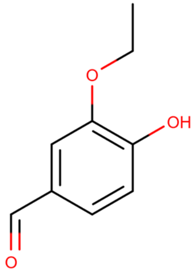	Ethyl vanillin	69.4	-	-	-	[22]
42	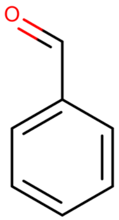	Benzaldedyde	3.0	-	-	-	[22]

43	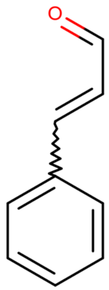	Cinnamaldehyde	1.1<br><br>6.1	-<br><br>27.2	-<br><br>-	-<br><br>41.1±3.5	[22]<br><br>[23]
44	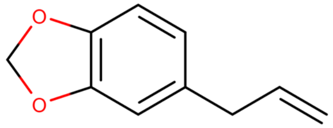	Safrole	12.0±0.5	30.9±3.6	-	14±4	[24]
45	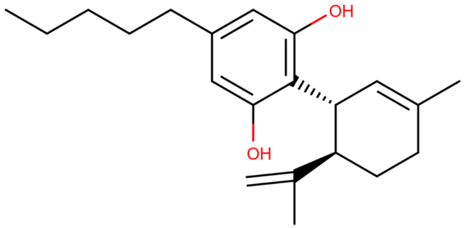	Cannabidiol (CBD)	0.27 ± 0.060[b]<br>0.23 ± 0.14[c]<br>0.21 ± 0.14[d]	-	-	-	[25]
46	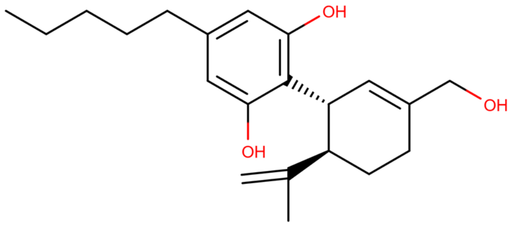	7-Hydroxycannabidiol<br><br>(7-OH-CBD)	0.45±0.18[b]<br>0.16±0.08[c]<br>0.78±0.23[d]	-	-	-	[25]

47	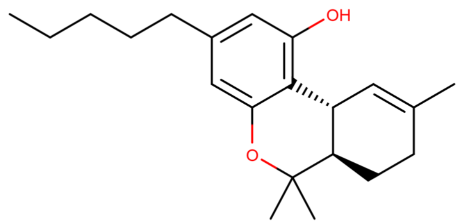	Δ9-tetrahydrocannabinol (THC)	16.8	0.862	-	-	[26]
48	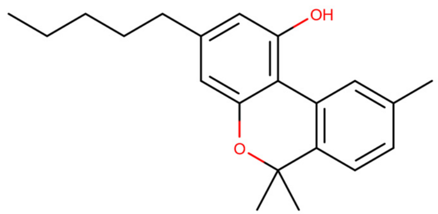	cannabinol (CBN)	10.4	1.01			[26]
49	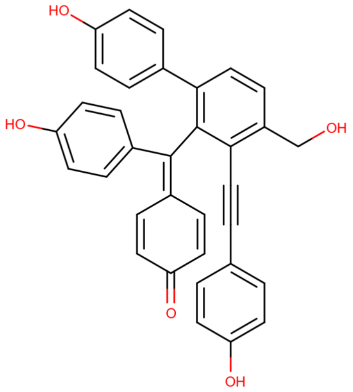	Selaginellin	>50[e]	-	-	-	[27]

50	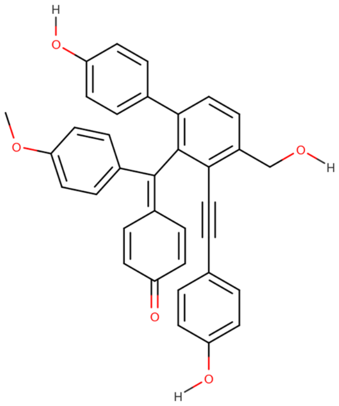	Selaginellin M	>50[e]	-	-	-	[27]
51	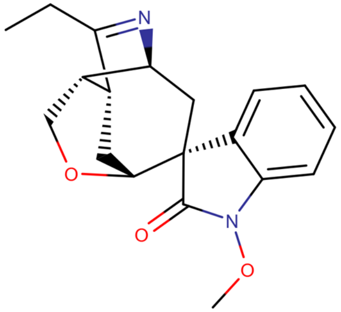	Humantenmine	45.87±5.29	-	-	-	[28]
52	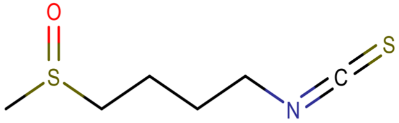	Sulforaphane	12	-	-	-	[29]

53	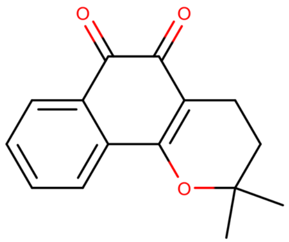	b-Lapachone	8.5	-	-	-	[30]
54	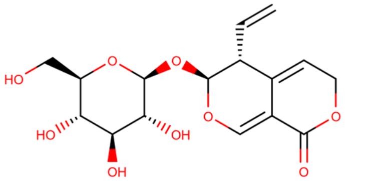	gentiopicroside	21.8[f]	8.12[f]	-	-	[31]
55	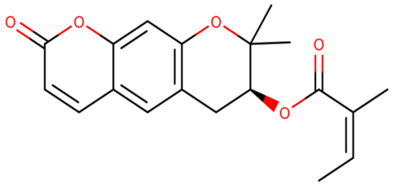	Decursinol angelate	4.4	0.996	-	~20	[32]
56	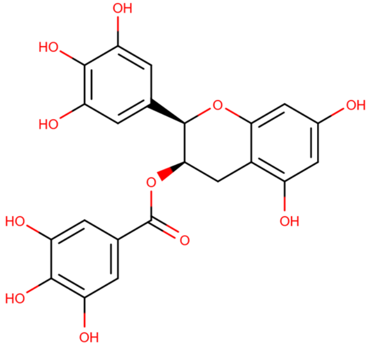	epigallocatechin gallate/ EGCG	-	41.1	-	-	[33]

57	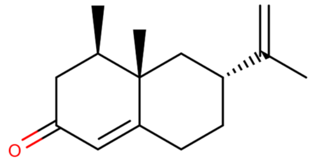	sesquiterpene/ nootkatone	11.5±4.9	0.8	-	-	[34]
58	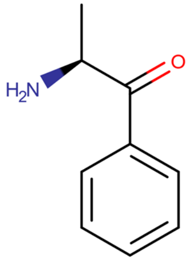	Cathinone	11.53	13.75	-	-	[35]

[a]The value reported by Endo *et.al* (2007)[8] and Pelkonen *et.al* (2008)[4] refers to the K_m constant of pilocarpine for CYP2A6
[b]Inhibition of nicotine metabolism to cotinine
[c]Inhibition of nicotine metabolism to nornicotine
[d]Inhibition of cotinine metabolism to trans-3′-hydroxycotinine (3HC)
[e]According to Heo *et.al* (2017) [27] the compounds showed low inhibitory potential
[f]Inhibition values are reported in units of μg/ml for the compounds in [31]

References

[1] Von Weymarn LB, Chun JA, Hollenberg PF. Effects of benzyl and phenethyl isothiocyanate on P450s 2A6 and 2A13: potential for chemoprevention in smokers. Carcinogenesis 2006;27:782–90. https://doi.org/10.1093/carcin/bgi301.

[2] Stephens ES, Walsh AA, Scott EE. Evaluation of Inhibition Selectivity for Human Cytochrome P450 2A Enzymes. Drug Metab Dispos 2012;40:1797–802. https://doi.org/10.1124/dmd.112.045161.

[3] Kramlinger VM, Von Weymarn LB, Murphy SE. Inhibition and inactivation of cytochrome P450 2A6 and cytochrome P450 2A13 by menthofuran, β-nicotyrine and menthol. Chem Biol Interact 2012;197:87–92. https://doi.org/10.1016/j.cbi.2012.03.009.

[4] Pelkonen O, Turpeinen M, Hakkola J, Honkakoski P, Hukkanen J, Raunio H. Inhibition and induction of human cytochrome P450 enzymes: current status. Arch Toxicol 2008;82:667–715. https://doi.org/10.1007/s00204-008-0332-8.

[5] Draper AJ, Madan A, Parkinson A. Inhibition of Coumarin 7-Hydroxylase Activity in Human Liver Microsomes. Arch Biochem Biophys 1997;341:47–61. https://doi.org/10.1006/abbi.1997.9964.

[6] Higashi E, Fukami T, Itoh M, Kyo S, Inoue M, Yokoi T, et al. Human CYP2A6 Is Induced by Estrogen via Estrogen Receptor. Drug Metab Dispos 2007;35:1935–41. https://doi.org/10.1124/dmd.107.016568.

[7] Higashi E, Nakajima M, Katoh M, Tokudome S, Yokoi T. Inhibitory Effects of Neurotransmitters and Steroids on Human CYP2A6. Drug Metab Dispos 2007;35:508–14. https://doi.org/10.1124/dmd.106.014084.

[8] Kinonen T, Pasanen M, Gynther J, Poso A, Järvinen T, Alhava E, et al. Competitive inhibition of coumarin 7-hydroxylation by pilocarpine and its interaction with mouse CYP 2A5 and human CYP $_2$ A6. Br J Pharmacol 1995;116:2625–30. https://doi.org/10.1111/j.1476-5381.1995.tb17217.x.

[9] Endo T, Ban M, Hirata K, Yamamoto A, Hara Y, Momose Y. Involvement of CYP2A6 in the Formation of a Novel Metabolite, 3-Hydroxypilocarpine, from Pilocarpine in Human Liver Microsomes. Drug Metab Dispos 2007;35:476–83. https://doi.org/10.1124/dmd.106.013425.

[10] Pouyfung P, Prasopthum A, Sarapusit S, Srisook E, Rongnoparut P. Mechanism-based Inactivation of Cytochrome P450 2A6 and 2A13 by Rhinacanthus nasutus Constituents. Drug Metab Pharmacokinet 2014;29:75–82. https://doi.org/10.2133/dmpk.DMPK-13-RG-048.

[11] Tiong KH, Mohammed Yunus NA, Yiap BC, Tan EL, Ismail R, Ong CE. Inhibitory Potency of 8-Methoxypsoralen on Cytochrome P450 2A6 (CYP2A6) Allelic Variants CYP2A6*15, CYP2A6*16, CYP2A6*21 and CYP2A6*22: Differential Susceptibility Due to Different Sequence Locations of the Mutations. PLoS ONE 2014;9:e86230. https://doi.org/10.1371/journal.pone.0086230.

[12] Boonruang S, Prakobsri K, Pouyfung P, Srisook E, Prasopthum A, Rongnoparut P, et al. Inhibition of human cytochromes P450 2A6 and 2A13 by flavonoids, acetylenic thiophenes and sesquiterpene lactones from *Pluchea indica* and *Vernonia cinerea*. J Enzyme Inhib Med Chem 2017;32:1136–42. https://doi.org/10.1080/14756366.2017.1363741.

[13] Prasopthum A, Pouyfung P, Sarapusit S, Srisook E, Rongnoparut P. Inhibition effects of Vernonia cinerea active compounds against cytochrome P450 2A6 and human monoamine oxidases, possible targets for reduction of tobacco dependence. Drug Metab Pharmacokinet 2015;30:174–81. https://doi.org/10.1016/j.dmpk.2014.12.005.

[14] Bojić M, Kondža M, Rimac H, Benković G, Maleš Ž. The Effect of Flavonoid Aglycones on the CYP1A2, CYP2A6, CYP2C8 and CYP2D6 Enzymes Activity. Molecules 2019;24:3174. https://doi.org/10.3390/molecules24173174.

[15] Tiong KH, Yiap BC, Tan EL, Ismail R, Ong CE. *In vitro* modulation of naturally occurring flavonoids on cytochrome P450 2A6 (CYP2A6) activity. Xenobiotica 2010;40:458–66. https://doi.org/10.3109/00498251003786749.

[16] Boonruang S, Prakobsri K, Pouyfung P, Prasopthum A, Rongnoparut P, Sarapusit S. Structure–activity relationship and *in vitro* inhibition of human cytochrome CYP2A6 and CYP2A13 by flavonoids. Xenobiotica 2020;50:630–9. https://doi.org/10.1080/00498254.2019.1675101.
[17] Koenigs LL, Trager WF. Mechanism-Based Inactivation of P450 2A6 by Furanocoumarins. Biochemistry 1998;37:10047–61. https://doi.org/10.1021/bi980003c.
[18] Ueng Y, Chen C, Chung Y, Liu T, Chang Y, Lo W, et al. Mechanism-based inhibition of cytochrome P450 (CYP)2A6 by chalepensin in recombinant systems, in human liver microsomes and in mice *in vivo*. Br J Pharmacol 2011;163:1250–62. https://doi.org/10.1111/j.1476-5381.2011.01341.x.
[19] Jeong S, Woo MM, Flockhart DA, Desta Z. Inhibition of drug metabolizing cytochrome P450s by the aromatase inhibitor drug letrozole and its major oxidative metabolite 4,4′-methanol-bisbenzonitrile in vitro. Cancer Chemother Pharmacol 2009;64:867–75. https://doi.org/10.1007/s00280-009-0935-7.
[20] Qi X, Dou T, Wang Z, Wu J, Yang L, Zeng S, et al. Inhibition of human cytochrome P450 2A6 by 7-hydroxycoumarin analogues: Analysis of the structure-activity relationship and isoform selectivity. Eur J Pharm Sci 2019;136:104944. https://doi.org/10.1016/j.ejps.2019.05.022.
[21] Fujita K, Kamataki T. Screening of organosulfur compounds as inhibitors of human CYP2A6. Drug Metab Dispos Biol Fate Chem 2001;29:983–9.
[22] Winters BR, Kochar TK, Clapp PW, Jaspers I, Madden MC. Impact of E-Cigarette Liquid Flavoring Agents on Activity of Microsomal Recombinant CYP2A6, the Primary Nicotine-Metabolizing Enzyme. Chem Res Toxicol 2020;33:1689–97. https://doi.org/10.1021/acs.chemrestox.9b00514.
[23] Chan J, Oshiro T, Thomas S, Higa A, Black S, Todorovic A, et al. Inactivation of CYP2A6 by the Dietary Phenylpropanoid trans-Cinnamic Aldehyde (Cinnamaldehyde) and Estimation of Interactions with Nicotine and Letrozole. Drug Metab Dispos 2016;44:534–43. https://doi.org/10.1124/dmd.115.067942.
[24] Ueng Y-F, Hsieh C-H, Don M-J. Inhibition of human cytochrome P450 enzymes by the natural hepatotoxin safrole. Food Chem Toxicol 2005;43:707–12. https://doi.org/10.1016/j.fct.2005.01.008.
[25] Nasrin S, Coates S, Bardhi K, Watson C, Muscat JE, Lazarus P. Inhibition of Nicotine Metabolism by Cannabidiol (CBD) and 7-Hydroxycannabidiol (7-OH-CBD). Chem Res Toxicol 2023;36:177–87. https://doi.org/10.1021/acs.chemrestox.2c00259.
[26] Yamaori S, Maeda C, Yamamoto I, Watanabe K. Differential inhibition of human cytochrome P450 2A6 and 2B6 by major phytocannabinoids. Forensic Toxicol 2011;29:117–24. https://doi.org/10.1007/s11419-011-0112-7.
[27] Heo J-K, Nguyen P-H, Kim W, Phuc N, Liu K-H. Inhibitory Effect of Selaginellins from Selaginella tamariscina (Beauv.) Spring against Cytochrome P450 and Uridine 5′-Diphosphoglucuronosyltransferase Isoforms on Human Liver Microsomes. Molecules 2017;22:1590. https://doi.org/10.3390/molecules22101590.
[28] Wang Y, Wu S, Chen Z, Zhang H, Zhao W. Inhibitory effects of cytochrome P450 enzymes CYP1A2, CYP2A6, CYP2E1 and CYP3A4 by extracts and alkaloids of Gelsemium elegans roots. J Ethnopharmacol 2015;166:66–73. https://doi.org/10.1016/j.jep.2015.03.002.
[29] Srovnalova A, Vanduchova A, Svecarova M, Anzenbacherova E, Tomankova V, Anzenbacher P, et al. Effects of sulforaphane and its S- and R-enantiomers on the expression and activities of human drug-metabolizing cytochromes P450. J Funct Foods 2015;14:487–501. https://doi.org/10.1016/j.jff.2015.02.006.
[30] Kim IS, Kim Y, Kwak TH, Yoo HH. Effects of β-lapachone, a new anticancer candidate, on cytochrome P450-mediated drug metabolism. Cancer Chemother Pharmacol 2013;72:699–702. https://doi.org/10.1007/s00280-013-2230-x.
[31] Deng Y, Wang L, Yang Y, Sun W, Xie R, Liu X, et al. In Vitro Inhibition and Induction of Human Liver Cytochrome P450 Enzymes by Gentiopicroside: Potent Effect on

CYP2A6. Drug Metab Pharmacokinet 2013;28:339–44. https://doi.org/10.2133/dmpk.DMPK-12-RG-090.
[32] Yoo HH, Lee MW, Kim YC, Yun C-H, Kim D-H. Mechanism-Based Inactivation of Cytochrome P450 2A6 by Decursinol Angelate Isolated from *Angelica Gigas*. Drug Metab Dispos 2007;35:1759–65. https://doi.org/10.1124/dmd.107.016584.
[33] Muto S, Fujita K, Yamazaki Y, Kamataki T. Inhibition by green tea catechins of metabolic activation of procarcinogens by human cytochrome P450. Mutat Res Mol Mech Mutagen 2001;479:197–206. https://doi.org/10.1016/S0027-5107(01)00204-4.
[34] Tassaneeyakul W, Guo L-Q, Fukuda K, Ohta T, Yamazoe Y. Inhibition Selectivity of Grapefruit Juice Components on Human Cytochromes P450. Arch Biochem Biophys 2000;378:356–63. https://doi.org/10.1006/abbi.2000.1835.
[35] Lim SYM, Loo JSE, Alshagga M, Alshawsh MA, Ong CE, Pan Y. In vitro and In silico studies of interactions of cathinone with human recombinant cytochrome P450 CYP(1A2), CYP2A6, CYP2B6, CYP2C8, CYP2C19, CYP2E1, CYP2J2, and CYP3A5. Toxicol Rep 2022;9:759–68. https://doi.org/10.1016/j.toxrep.2022.03.040.
